# Supplementary material for: Diagnostic Practices and Treatment for P. vivax in the InterEthnic Therapeutic Encounter of South-Central Vietnam: A Mixed-Methods Study
Source: Pathogens. 2020 Dec 31;10(1):26. doi: 10.3390/pathogens10010026 (PMC7824694; doi:10.3390/pathogens10010026)
Supplement: Supplementary file 1 [file pathogens-10-00026-s001.pdf]

Supplementary 1. Treatment guidelines for non-complicated malaria[i]

Table 1. Malaria treatment by age group and malaria species

**Age group**	**Clinical malaria**	***P. falciparum* malaria**	***P. vivax* or *P. ovale* malaria**	***P. malariae* or *P. knowlesi* malaria**	**Mixed infection including *P. falciparum***
Under 6 months of age	DHA-PPQ[ii]	DHA-PPQ	CQ[iii]	CQ	DHA-PPQ
From 6 months and older	DHA-PPQ	DHA-PPQ + PQ or other combined therapy	CQ + PQ[iv]	CQ+PQ	DHA-PPQ + PQ or other combined therapy
Pregnant women in the first trimester	Quinine + Clindamycin	Quinine + Clindamycin	CQ	CQ	Quinin + Clindamycin
Pregnant women in the 2nd and 3rd trimester	DHA-PPQ	DHA-PPQ + PQ or other combined therapy	CQ	CQ	DHA-PPQ + PQ or other combined therapy

First line treatment:

- P. falciparum: DHA – PPQ in 3 days and one dosage of PQ 0.5mg/kg base
- Mixed infections including *P. falciparum*: DHA-PPQ in 3 days and PQ 0.25mg/kg base in 14 days

- *P. vivax* or *P. ovale*: CQ in 3 days and one dosage of PQ 0.25mg/kg base in 14 days
- *P. malariae* or *P. knowlesi*: CQ in 3 days and one dosage of PQ 0.5mg/kg base

Table 2. Dihydroartemisinin dosage (each tablet contains 40mg Dihydroartemisinin and 320mg Piperaquine phosphate) by weight[v]

Weight	Day 1	Day 2	Day 3
<8kg	½ tablet	½ tablet	½ tablet
8 to <17kg	1 tablet	1 tablet	1 tablet
17 to <25kg	1 ½ tablets	1 ½ tablets	1 ½ tablets
25 to <36kg	2 tablets	2 tablets	2 tablets
36 to <60kg	3 tablets	3 tablets	3 tablets
≥60kg	4 tablets	4 tablets	4 tablets

Table 3. Chloroquine dosage (each tablet contains 250mg Chloroquine) by age group

Age group	Day 1	Day 2	Day 3
Under 1 year	½ tablets	½ tablets	½ tablets
1 to 5 years	1 tablet	1 tablet	1 tablet
5 to under 12 years	2 tablets	2 tablets	2 tablets
12 to under 15 years	3 tablets	3 tablets	3 tablets
15 years and older	4 tablets	4 tablets	4 tablets

CQ dosage can be adjusted by weight, the total dosage is 25mg/kg base. In the first 2-day, the total dosage is divided into two, with 6 hours interval between each dosage. In day 3, only one dosage is applied. Daily dosage is following:

- Day 1: 10mg/kg base
- Day 2: 10mg/kg base

- Day 3: 5mg/kg base

Table 4. Quinine sulfate dosage (each tablet contains 250mg) by age group

**Age group**	**Tablet x days**	**Note**
Under 1 year	1 tablet/day x 7 days	Divide daily dosage in 3
1 to 5 years	1 ½ tablets x 7 days	
5 to under 12 years	3 tablets/day x 7 days	
12 to under 15 years	5 tablets/day x 7 days	
15 years and older	6 tablets/day x 7 days	

Quinine sulfate dosage can be adjusted by weight, using 30mg/kg base/24 hours (divided in 3 times per day) in 7 days.

Table 5. Primaquine dosage (each tablet of 13.2mg contains 7.5mg PQ) by age group

**Age group**	***P. falciparum / P. Knowles / P. malariae***	***P. vivax / P. ovale***
6 months old to 3 years	½ tablets – 1 time	½ tablet in 14 days
3 to 5 years	1 tablet – 1 time	1 tablet in 14 days
5 to under 12 years	2 tablets – 1 time	2 tablets in 14 days
12 to under 15 years	3 tablets – 1 time	3 tablets in 14 days
15 years and older	4 tablets – 1 time	4 tablets in 14 days

PQ dosage can be adjusted by weight.

- Treatment for gametocytes in patients infected by *P. falciparum* /*P. malariae* / *P. knowlesi*: one dosage of 0.5mg base/kg in the 3rd day of treatment.
- Treatment for patients infected by *P. vivax* or *P. ovale:* 0.25mg base/kg/day in 14 days, starting in the first day of treatment together with CQ to treat sporozoite in prevention of relapse.

Notes:

- Do not prescribe PQ for children under six month-old and pregnant women, women who breastfeed children < 6 month-old and patients with liver disease. If there is no testing to determine G6PD deficiency, it is needed to monitor red cells, skin colour and urine colour and stop PQ in case urine colour is darker, similar to the colour of tea or black coffee.
- For *P. vivax* or *P. ovale* patients who have G6PD deficiency, recommended PQ dosage is 0.75mg base/kg per week in 8 weeks. It is needed to closely monitor the patient during treatment period.
- PQ should be taken after meal.

Supplementary 2. Treatment for hemoglobinuria

The signs and symptoms of hemoglobinuria are clear malaria clinical symptoms, yellowish skin, and dark urine colour (similar to coffee or dark tea colour). It is needed to ask the patients about history of hemoglobinuria, recent medicine use, blood test to determine malaria infection, urine test to count haemoglobin and multiple tests to see if there is a rapid decline of red cells. If it is possible, proceed to G6PD testing.

Treatment guidelines:

+ Transfusion of 0.9% NaCl (normal saline) and fluids to maintain urine excretion of ≥ 2500 ml/24 hours in adult patient or 10-12 ml/kg/24 hours in children patient.
+ Transfusion of red cells when Hematocrit is < 25% or hemoglobin is < 7g/dl.
+ If signs of hemoglobinuria occur in patient who is on Primaquin or Quinin, immediately terminate these medicines and change to other antimalarials.
+ If patient has kidney failure, apply treatment guidelines as for acute malaria case.

Notes: signs of hemoglobinuria often occur amongst patients whose G6PD is deficient, or when oxidization occurs due to taking some medicine, food or due to an infection. It is needed to check

carefully the history of patient, conduct several blood tests to determine hemoglobinuria due to malaria parasite as well as to eliminate other causations of hemoglobinuria.

---

[i] Ministry of Health of Vietnam. Decision 4845/QD-BYT on Guidelines on diagnosis and treatment for malaria. Hanoi, 8 September 2016

[ii] Dihydroartemisinin – Piperaquine phosphate. Commercial names are Artecan, Arterakine

[iii] Chloroquine

[iv] Primaquine

[v] Does not apply for pregnant women in their first trimester
